# Bis[4-(2-aza­niumyleth­yl)piperazin-1-ium] di-μ-sulfido-bis­[disulfido­germanate(II)]

**DOI:** 10.1107/S1600536812022040

**Published:** 2012-05-26

**Authors:** Bei-Bei Zhang, Chao Xu, Taike Duan, Qun Chen, Qian-Feng Zhang

**Affiliations:** a Institute of Molecular Engineering and Applied Chemsitry, Anhui University of Technology, Ma’anshan, Anhui 243002, People’s Republic of China; b Department of Applied Chemistry, School of Petrochemical Engineering, Changzhou University, Jiangsu 213164, People’s Republic of China

## Abstract

In the title compound, (C_6_H_17_N_3_)_2_[Ge_2_S_6_], the dimeric [Ge_2_S_6_]^4−^ anion is formed by two edge-sharing GeS_4_ tetra­hedral units. The average terminal and bridging Ge—S bond lengths are 2.164 (2) and 2.272 (8) Å, respectively. The dimeric inorganic anions and the organic piperazinium cations are organized into a three-dimensional network by N—H⋯S hydrogen bonds.

## Related literature

For background to main-group metal–chalcogenide compounds, see: Bedard *et al.* (1989 ▶); Yaghi *et al.* (1994 ▶); Bowes & Ozin (1996 ▶); Zheng *et al.* (2005 ▶); Zhang *et al.* (2008 ▶); Haddadpour *et al.* (2009 ▶). For related structures, see: Jia *et al.* (2005 ▶); Xu *et al.* (2012 ▶).
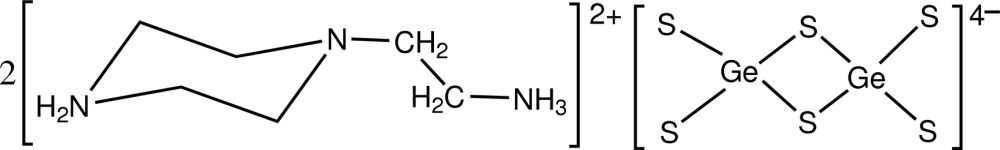


## Experimental

### 

#### Crystal data

(C_6_H_17_N_3_)_2_[Ge_2_S_6_]
*M* *_r_* = 600.10
Triclinic, 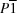

*a* = 7.4985 (1) Å
*b* = 8.2709 (1) Å
*c* = 10.4177 (1) Å
α = 72.156 (1)°
β = 78.323 (1)°
γ = 89.792 (1)°
*V* = 601.11 (1) Å^3^
*Z* = 1
Mo *K*α radiation
μ = 3.03 mm^−1^
*T* = 296 K
0.21 × 0.16 × 0.13 mm

#### Data collection

Bruker APEXII CCD area-detector diffractometer
Absorption correction: multi-scan (*SADABS*; Sheldrick, 1997 ▶) *T*_min_ = 0.568, *T*_max_ = 0.694
11264 measured reflections
2756 independent reflections
2558 reflections with *I* > 2σ(*I*)
*R*_int_ = 0.018

#### Refinement

*R*[*F* ^2^ > 2σ(*F* ^2^)] = 0.017
*wR*(*F* ^2^) = 0.044
*S* = 1.04
2756 reflections
118 parameters
H-atom parameters constrained
Δρ_max_ = 0.34 e Å^−3^
Δρ_min_ = −0.20 e Å^−3^

### 

Data collection: *APEX2* (Bruker, 2005 ▶); cell refinement: *SAINT* (Bruker, 2005 ▶); data reduction: *SAINT*; program(s) used to solve structure: *SHELXS97* (Sheldrick, 2008 ▶); program(s) used to refine structure: *SHELXL97* (Sheldrick, 2008 ▶); molecular graphics: *SHELXTL* (Sheldrick, 2008 ▶); software used to prepare material for publication: *SHELXTL*.

## Supplementary Material

Crystal structure: contains datablock(s) I, global. DOI: 10.1107/S1600536812022040/mw2068sup1.cif

Structure factors: contains datablock(s) I. DOI: 10.1107/S1600536812022040/mw2068Isup2.hkl

Additional supplementary materials:  crystallographic information; 3D view; checkCIF report

## Figures and Tables

**Table 1:** Hydrogen-bond geometry (Å, °)

*D*—H⋯*A*	*D*—H	H⋯*A*	*D*⋯*A*	*D*—H⋯*A*
N1—H1*B*⋯S2^i^	0.90	2.50	3.3864 (14)	170
N1—H1*A*⋯S2^ii^	0.90	2.49	3.2990 (14)	150
N2—H2*A*⋯S3	0.89	2.43	3.2781 (13)	160
N2—H2*C*⋯S2^iii^	0.89	2.51	3.3467 (13)	157
N2—H2*B*⋯S3^iv^	0.89	2.42	3.3021 (13)	172

Symmetry codes: (i) 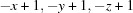; (ii) 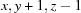; (iii) 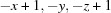; (iv) 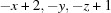.

**Fig. 1.**
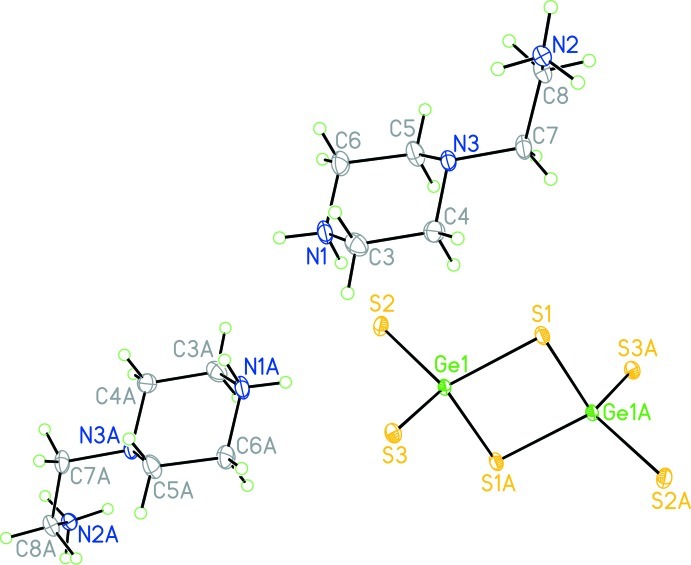
Perspective view of the title compound with displacement ellipsoids at the 50% probability level.

**Fig. 2.**
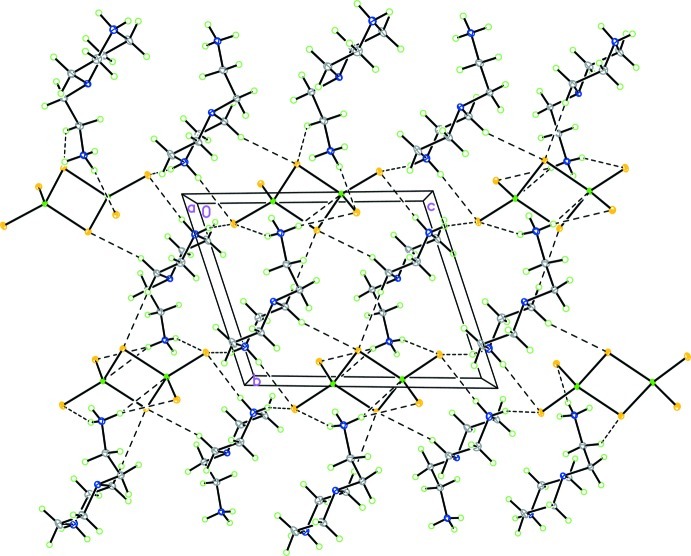
Packing diagram of the title compound. Dashed lines denote hydrogen bonds.

## supplementary crystallographic information

### Comment

Main group (groups 13 and 14) metal chalcogenide complexes with well-defined compositions and structures are of great interest because of their size-dependent optical properties, semiconducting and photocatalytic behaviors (Bowes & Ozin, 1996; Zhang *et al.*, 2008). Since Bedard synthesized the first porous chalcogenide compound by replacing O^2-^ with S^2-^ in an open-framework oxide (Bedard *et al.*, 1989), much effort has been expended during the past two decades to develop open-framework metal chalcogenides (Yaghi *et al.*, 1994; Zheng *et al.*, 2005; Haddadpour *et al.*, 2009). In this paper we report the hydrothermal synthesis and crystal structure of a new thiogermanate, [aepH_2_]_2_[Ge_2_S_6_]. (aep = *N*-(2-aminoethyl)piperazinium).

The title compound consists of a dimeric [Ge_2_S_6_]^4-^ anion having crystallographically-imposed centrosymmetry and two diprotonated [aepH_2_]^2+^ cations (Fig. 1). The dimeric [Ge_2_S_6_]^4-^ anion is constructed by two edge-linked tetrahedral GeS_4_ units forming a planar Ge_2_S_2_ quadrilateral while the four terminal sulfur atoms lie in a second plane at an angle of 88.38 (1)° to the first. The S—Ge—S angles in the tetrahedral GeS_4_ unit range from 91.827 (13) to 114.634 (15)°. The average Ge—S_t_ (terminal bond) length of 2.1642 (4) Å) is significantly shorter than the average Ge—S_b_ (bridging bond) length of 2.2724 (4) Å) with both values similar to those found in the other thiogermanates (Xu *et al.*, 2012; Jia *et al.*, 2005). The two terminal amine groups of the *N*-(2-aminoethyl)piperazine are protonated to balance the negative charge of the dimeric anion. The [Ge_2_S_6_]^4-^ anions and [apeH_2_]^2+^ cations are organized into an extended three-dimensional network by N—H···S hydrogen bonds (Fig. 2 and Table 1).

### Experimental

GeO_2_ (104.6 mg, 1.0 mmol) and S powder (128.0 mg, 4.0 mmol) were mixed with *N*-(2-aminoethyl)piperazine (2.3478 g) in a 23 mL Teflon-lined stainless steel autoclave and stirred for 20 min. The vessel was sealed and heated at 190°C for 7 d and then cooled to room temperature. Colorless slab crystals were obtained and air dried. The yield based on GeO_2_ is about 36%. Analysis, calculated for C_12_H_34_N_6_S_6_Ge_2_: C 24.0, H 5.71, N 14.0%; found C 23.7, H 5.56, N 13.9%.

### Refinement

The structure was solved by direct methods and refined by full-matrix least-squares methods based on F^2^. All C-bound H atoms were positioned and refined as riding atoms with C—H = 0.97(CH_2_) Å and *U*_iso_(H) = 1.2*U*_eq_(C). N-bound H atoms were located in a difference map, adjusted to give N—H = 0.90 Å and refined as riding atoms with *U*_iso_(H) = 1.2*U*_eq_(N).

### Crystal data

**Table:**

(C_6_H_17_N_3_)_2_[Ge_2_S_6_]	*Z* = 1
*M**_r_* = 600.10	*F*(000) = 308
Triclinic, *P*1	*D*_x_ = 1.657 Mg m^−^^3^
Hall symbol: -P 1	Mo *K*α radiation, λ = 0.71073 Å
*a* = 7.4985 (1) Å	Cell parameters from 6350 reflections
*b* = 8.2709 (1) Å	θ = 2.5–26.4°
*c* = 10.4177 (1) Å	µ = 3.03 mm^−^^1^
α = 72.156 (1)°	*T* = 296 K
β = 78.323 (1)°	Slab, colorless
γ = 89.792 (1)°	0.21 × 0.16 × 0.13 mm
*V* = 601.11 (1) Å^3^	

### Data collection

**Table:**

Bruker APEXII CCD area-detector diffractometer	2756 independent reflections
Radiation source: fine-focus sealed tube	2558 reflections with *I* > 2σ(*I*)
Graphite monochromator	*R*_int_ = 0.018
φ and ω scans	θ_max_ = 27.5°, θ_min_ = 2.1°
Absorption correction: multi-scan (*SADABS*; Sheldrick, 1997)	*h* = −9→9
*T*_min_ = 0.568, *T*_max_ = 0.694	*k* = −10→10
11264 measured reflections	*l* = −13→13

### Refinement

**Table:**

Refinement on *F*^2^	Primary atom site location: structure-invariant direct methods
Least-squares matrix: full	Secondary atom site location: difference Fourier map
*R*[*F*^2^ > 2σ(*F*^2^)] = 0.017	Hydrogen site location: inferred from neighbouring sites
*wR*(*F*^2^) = 0.044	H-atom parameters constrained
*S* = 1.04	*w* = 1/[σ^2^(*F*_o_^2^) + (0.0255*P*)^2^ + 0.0568*P*] where *P* = (*F*_o_^2^ + 2*F*_c_^2^)/3
2756 reflections	(Δ/σ)_max_ = 0.001
118 parameters	Δρ_max_ = 0.34 e Å^−^^3^
0 restraints	Δρ_min_ = −0.20 e Å^−^^3^

### Special details

**Table:**

Geometry. All esds (except the esd in the dihedral angle between two l.s. planes) are estimated using the full covariance matrix. The cell esds are taken into account individually in the estimation of esds in distances, angles and torsion angles; correlations between esds in cell parameters are only used when they are defined by crystal symmetry. An approximate (isotropic) treatment of cell esds is used for estimating esds involving l.s. planes.
Refinement. Refinement of F^2^ against ALL reflections. The weighted R-factor wR and goodness of fit S are based on F^2^, conventional R-factors R are based on F, with F set to zero for negative F^2^. The threshold expression of F^2^ > 2sigma(F^2^) is used only for calculating R-factors(gt) etc. and is not relevant to the choice of reflections for refinement. R-factors based on F^2^ are statistically about twice as large as those based on F, and R- factors based on ALL data will be even larger.

### Fractional atomic coordinates and isotropic or equivalent isotropic displacement parameters (Å^2^)

**Table:**

	*x*	*y*	*z*	*U*_iso_*/*U*_eq_	
Ge1	0.534452 (18)	−0.015013 (17)	0.647883 (13)	0.02329 (5)	
S1	0.39310 (5)	0.17306 (4)	0.49657 (4)	0.03133 (9)	
S2	0.34914 (5)	−0.14470 (5)	0.84118 (4)	0.03350 (9)	
S3	0.77604 (5)	0.09503 (5)	0.68057 (4)	0.03090 (9)	
N1	0.6536 (2)	0.80969 (18)	0.03620 (14)	0.0430 (3)	
H1A	0.6097	0.8421	−0.0415	0.052*	
H1B	0.6380	0.8934	0.0751	0.052*	
N2	0.94141 (17)	0.20541 (15)	0.34735 (13)	0.0330 (3)	
H2A	0.8730	0.1634	0.4320	0.049*	
H2B	1.0236	0.1318	0.3323	0.049*	
H2C	0.8707	0.2230	0.2859	0.049*	
N3	0.82332 (17)	0.56300 (15)	0.22444 (12)	0.0310 (3)	
C3	0.5509 (2)	0.6512 (2)	0.1331 (2)	0.0471 (4)	
H3A	0.5581	0.5636	0.0884	0.057*	
H3B	0.4234	0.6728	0.1586	0.057*	
C4	0.6293 (2)	0.5917 (2)	0.25953 (17)	0.0404 (4)	
H4A	0.6139	0.6763	0.3074	0.048*	
H4B	0.5638	0.4867	0.3212	0.048*	
C5	0.9224 (2)	0.7215 (2)	0.13193 (18)	0.0462 (4)	
H5A	1.0512	0.7030	0.1096	0.055*	
H5B	0.9088	0.8079	0.1780	0.055*	
C6	0.8514 (3)	0.7828 (3)	0.00124 (18)	0.0513 (5)	
H6A	0.9172	0.8886	−0.0584	0.062*	
H6B	0.8699	0.6992	−0.0475	0.062*	
C7	0.9006 (3)	0.5008 (2)	0.34801 (17)	0.0416 (4)	
H7A	0.8031	0.4515	0.4279	0.050*	
H7B	0.9606	0.5955	0.3625	0.050*	
C8	1.0363 (2)	0.3689 (2)	0.33375 (18)	0.0384 (3)	
H8A	1.1192	0.4095	0.2445	0.046*	
H8B	1.1075	0.3511	0.4044	0.046*	

### Atomic displacement parameters (Å^2^)

**Table:**

	*U*^11^	*U*^22^	*U*^33^	*U*^12^	*U*^13^	*U*^23^
Ge1	0.02519 (8)	0.02634 (8)	0.02008 (8)	0.00381 (5)	−0.00971 (5)	−0.00663 (6)
S1	0.0419 (2)	0.03052 (18)	0.02880 (19)	0.01500 (14)	−0.01897 (15)	−0.01251 (14)
S2	0.03277 (19)	0.0426 (2)	0.02247 (17)	−0.00458 (15)	−0.00702 (14)	−0.00547 (15)
S3	0.02968 (18)	0.03499 (19)	0.03217 (19)	0.00113 (14)	−0.01403 (14)	−0.01165 (15)
N1	0.0604 (9)	0.0426 (8)	0.0369 (7)	0.0181 (6)	−0.0304 (7)	−0.0153 (6)
N2	0.0350 (6)	0.0325 (6)	0.0308 (6)	0.0071 (5)	−0.0105 (5)	−0.0066 (5)
N3	0.0387 (7)	0.0290 (6)	0.0265 (6)	0.0061 (5)	−0.0144 (5)	−0.0056 (5)
C3	0.0397 (9)	0.0499 (10)	0.0598 (11)	0.0059 (7)	−0.0220 (8)	−0.0214 (9)
C4	0.0414 (9)	0.0377 (8)	0.0375 (9)	0.0026 (7)	−0.0057 (7)	−0.0067 (7)
C5	0.0395 (9)	0.0485 (10)	0.0411 (9)	−0.0035 (7)	−0.0160 (7)	0.0046 (8)
C6	0.0546 (11)	0.0568 (11)	0.0310 (9)	0.0061 (9)	−0.0104 (8)	0.0037 (8)
C7	0.0641 (11)	0.0337 (8)	0.0330 (8)	0.0120 (7)	−0.0255 (8)	−0.0097 (7)
C8	0.0391 (8)	0.0371 (8)	0.0404 (9)	0.0034 (6)	−0.0211 (7)	−0.0062 (7)

### Geometric parameters (Å, º)

**Table:**

Ge1—S3	2.1628 (4)	C3—C4	1.495 (2)
Ge1—S2	2.1658 (4)	C3—H3A	0.9700
Ge1—S1^i^	2.2668 (4)	C3—H3B	0.9700
Ge1—S1	2.2780 (4)	C4—H4A	0.9700
S1—Ge1^i^	2.2668 (4)	C4—H4B	0.9700
N1—C6	1.487 (2)	C5—C6	1.504 (2)
N1—C3	1.487 (2)	C5—H5A	0.9700
N1—H1A	0.9000	C5—H5B	0.9700
N1—H1B	0.9000	C6—H6A	0.9700
N2—C8	1.4845 (19)	C6—H6B	0.9700
N2—H2A	0.8900	C7—C8	1.509 (2)
N2—H2B	0.8900	C7—H7A	0.9700
N2—H2C	0.8900	C7—H7B	0.9700
N3—C4	1.464 (2)	C8—H8A	0.9700
N3—C5	1.4637 (19)	C8—H8B	0.9700
N3—C7	1.467 (2)		
			
S3—Ge1—S2	111.635 (15)	N3—C4—H4A	109.4
S3—Ge1—S1^i^	111.482 (15)	C3—C4—H4A	109.4
S2—Ge1—S1^i^	114.630 (15)	N3—C4—H4B	109.4
S3—Ge1—S1	113.476 (15)	C3—C4—H4B	109.4
S2—Ge1—S1	112.453 (16)	H4A—C4—H4B	108.0
S1^i^—Ge1—S1	91.835 (13)	N3—C5—C6	110.83 (14)
Ge1^i^—S1—Ge1	88.165 (13)	N3—C5—H5A	109.5
C6—N1—C3	110.87 (13)	C6—C5—H5A	109.5
C6—N1—H1A	109.4	N3—C5—H5B	109.5
C3—N1—H1A	109.5	C6—C5—H5B	109.5
C6—N1—H1B	109.5	H5A—C5—H5B	108.1
C3—N1—H1B	109.4	N1—C6—C5	109.21 (15)
H1A—N1—H1B	108.1	N1—C6—H6A	109.8
C8—N2—H2A	109.5	C5—C6—H6A	109.8
C8—N2—H2B	109.5	N1—C6—H6B	109.8
H2A—N2—H2B	109.5	C5—C6—H6B	109.8
C8—N2—H2C	109.5	H6A—C6—H6B	108.3
H2A—N2—H2C	109.5	N3—C7—C8	111.07 (13)
H2B—N2—H2C	109.5	N3—C7—H7A	109.4
C4—N3—C5	109.42 (13)	C8—C7—H7A	109.4
C4—N3—C7	111.63 (13)	N3—C7—H7B	109.4
C5—N3—C7	109.98 (13)	C8—C7—H7B	109.4
N1—C3—C4	109.82 (13)	H7A—C7—H7B	108.0
N1—C3—H3A	109.7	N2—C8—C7	110.74 (13)
C4—C3—H3A	109.7	N2—C8—H8A	109.5
N1—C3—H3B	109.7	C7—C8—H8A	109.5
C4—C3—H3B	109.7	N2—C8—H8B	109.5
H3A—C3—H3B	108.2	C7—C8—H8B	109.5
N3—C4—C3	111.20 (13)	H8A—C8—H8B	108.1

Symmetry code: (i) −*x*+1, −*y*, −*z*+1.

### Hydrogen-bond geometry (Å, º)

**Table:**

*D*—H···*A*	*D*—H	H···*A*	*D*···*A*	*D*—H···*A*
N1—H1*B*···S2^ii^	0.90	2.50	3.3864 (14)	170
N1—H1*A*···S2^iii^	0.90	2.49	3.2990 (14)	150
N2—H2*A*···S3	0.89	2.43	3.2781 (13)	160
N2—H2*C*···S2^i^	0.89	2.51	3.3467 (13)	157
N2—H2*B*···S3^iv^	0.89	2.42	3.3021 (13)	172

Symmetry codes: (i) −*x*+1, −*y*, −*z*+1; (ii) −*x*+1, −*y*+1, −*z*+1; (iii) *x*, *y*+1, *z*−1; (iv) −*x*+2, −*y*, −*z*+1.
